# Biological Staining and Culturing in Infectious Keratitis: Controversy in Clinical Utility

**Published:** 2019

**Authors:** Majid Moshirfar, Grant C. Hopping, Uma Vaidyanathan, Harry Liu, Anisha N. Somani, Yasmyne C. Ronquillo, Phillip C. Hoopes

**Affiliations:** 1 Hoopes Durrie Rivera Research Center, Hoopes Vision, Draper, UT, USA; 2 Utah Lions Eye Bank, Murray, UT, USA; 3 John A. Moran Eye Center, Department of Ophthalmology and Visual Sciences, University of Utah School of Medicine, Salt Lake City, UT, USA; 4 McGovern Medical School, University of Texas Health Science Center, Houston, TX, USA

**Keywords:** Keratitis, Corneal Ulcer, Culture, Biologic Stains, Sensitivity, Specificity, Polymerase Chain Reaction, Fungi, Acanthamoeba, Herpes Simplex, Bacteria, Antibiotic sensitivity testing

## Abstract

Infectious keratitis causes significant, financial burden and is only increasing in frequency with contact lens use. Despite this, no retrospective studies, prospective studies, or clinical trials have evaluated the diagnostic validity of clinical guidelines in cases of infectious keratitis. Currently, standard of care recommends that corneal samples be obtained for staining and culturing in select patients showing evidence of corneal ulceration. Ideally, diagnostic information from corneal sampling is thought to help guide therapeutic interventions, prevent disease progression, reduce antibiotic resistance, and decrease overall expenditures for the management and treatment of infectious keratitis. However, current staining and culturing methods are limited by poor sensitivity in non-bacterial cases (i.e. fungal, viral) and lengthy turnaround times, and these methods do not frequently change clinical decision making. Newer fluoroquinolones and broad-spectrum antibiotics resolve the vast majority of cases of infectious keratitis, rendering cultures less essential for management. We studied the clinical utility of obtaining corneal samples for culturing and staining and the need for future research to establish superior diagnostic guidelines for their use in infectious keratitis.

## INTRODUCTION

A far too common dilemma that cornea specialists and general ophthalmologists face is whether to culture corneal ulcers. Infectious keratitis is characterized by inflammation of the cornea due to microbial infiltration, often accompanied by a loss of stroma and disrupted epithelium [1, 2]. Patients with infectious keratitis can present with rapid-onset pain, blurred vision, mucopurulent discharge, and photophobia [3]. Microbial infection by bacteria, fungi, viruses, and parasites are all causes of infectious keratitis, which can quickly progress to ulceration, corneal perforation, endophthalmitis, and scarring with subsequent loss of vision [4, 5]. An estimated 71,000 cases of ulcerative keratitis occur annually in the U.S. [6]; rising incidence has been associated with increasing contact lens use [7]. Clinicians spend an estimated 250,000 h managing patients with keratitis every year, and approximately 175 million USD is spent annually treating keratitis and related disorders, posing a significant strain on healthcare expenditures [8]. With the appearance of broad-spectrum fluoroquinolones and fortified antibiotics that clear most of these infections, practitioners may question the usefulness of collecting corneal scrapings for smears, cultures, and antibiotic sensitivity testing.

Corneal ulcers typically form after a break in the epithelium results in stromal infiltration by opportunistic pathogens [5]. Gram-positive bacteria (staphylococcal and streptococcal species) are the most common bacteria recovered in infectious keratitis. Pseudomonas aeruginosa is a frequent Gram-negative cause, especially in contact lens overuse [5, 9, 10]. However, regional estimates vary [9]. A lack of clinical response may lead a clinician to consider less common organisms, such as atypical mycobacteria, fungi (i.e., Aspergillus), viruses (i.e., herpes simplex virus [HSV]) and Acanthamoeba, as causative agents in refractory cases of infectious keratitis [10-12]. A significant minority of keratitis is polymicrobial in origin, making cultures and smears less useful for diagnosis due to the difficulty distinguishing causative pathogens from contamination [5, 13]. 

## METHODS

To find information on Biological Staining and Culturing in Infectious Keratitis, a literature search was performed using the following sources: PubMed, Google Scholar, Embase, and Scopus. Keywords used in this search included infectious keratitis, ulcerative keratitis, bacterial keratitis, microbial keratitis, staining, culture, smears, antibiotic sensitivity testing, treatment, sensitivity, specificity, polymerase chain reaction, fungal keratitis, Acanthamoeba, and herpetic keratitis. Articles describing infectious keratitis, culturing, and staining were systematically reviewed. Reference lists of these articles were used to identify additional articles. There were no language restrictions. Publications were drawn between the dates of 1980-2019.

**CURRENT STANDARD OF CARE**

The American Academy of Ophthalmology (AAO) has not released a preferred practice pattern specifically for infectious keratitis; however, AAO recently published the Bacterial Keratitis Preferred Practice Pattern, outlining their recommendations for the diagnosis and management in suspected cases of bacterial keratitis. It advises that smears and cultures be collected from ulcers (1) that are large (>2mm) and centrally located, (2) with significant stromal melting, (3) that are refractory to empiric antibiotic therapy, (4) in cases with prior history of corneal surgery, (5) with multiple, diffuse, stromal infiltrates, or (6) with atypical presentations, suggestive of amoebic, fungal, or mycobacterial infection [11].

Initial empiric therapies typically start with fluoroquinolone eyedrops. Alternatively, a fortified aminoglycoside can be used with a cephalosporin or vancomycin, depending on initial clinical severity. If refractive to empiric therapy and cultures are negative, repeat cultures of the ulcer and referral to a cornea specialist may be warranted (Table 1) [11]. Yet, no retrospective studies, prospective studies, or randomized clinical trials to date have evaluated the current clinical guidelines for the diagnosis of infectious keratitis, leaving much to clinical judgment. In a study of 436 ophthalmologists, respondents on average culture corneal ulcers in only 35% of cases [14]. About 13% of general ophthalmologists willing to treat a case of severe ulcerative keratitis would not obtain cultures before starting empiric treatment [15]. Well-designed prospective studies are needed to validate, and possibly improve, current published guidelines and unify discrepancies of clinical practice within the field of ophthalmology [14-16].

**Table 1:** Current Diagnostic and Therapeutic Guidelines for Infectious Keratitis

Causative Agent	Staining		Culture		Treatment
	Type	Sensitivity*	Type	Sensitivity*	
Bacterial	Gram, Giemsa [11]	57-67% [13, 17, 18]	Blood agar, Chocolate agar, Thioglycolate broth, Mannitol salt agar [11]	58% [19]	Fluoroquinolones *or *aminoglycoside + cephalosporins *or *aminoglycoside + vancomycin
Viral i.HSV1 ii. VZV iii. CMV	N/A	N/A	i.Vero cell line [20] ii. MRC-5 cell line [21] iii. MRC-5 cell line [22]	i.21% [20] ii. 46% [21]	i.Acyclovir (oral) + Trifluridine (topical), acyclovir (topical), valganciclovir ii.Acyclovir (oral), famciclovir (oral), valacyclovir (oral), acyclovir (topical) iii.Valganciclovir (oral), Valganciclovir (topical)
Fungal	KOH [23, 24]	68-99% [23, 24]	Sabouraud dextrose agar [11]	25-59% [24-27]	Natamycin, amphotericin, clotrimazole, fluconazole, itraconazole, voriconazole
Acanthamoeba	KOH + CFW [13]	84% [13]	Buffered charcoal yeast extract, Escherichia coli-seeded non-nutrient agar plates [11]	33% [28]	Chlorhexidine + PHMB *or *chlorhexidine + brolene *or *neomycin

Abbreviations: KOH: Potassium hydroxide; CFW: Calcofluor white; HSV1: herpes simplex virus type 1; VZV: varicella-zoster virus; CMV: cytomegalovirus; N/A: not available; MRC-5: human lung embryonated cells; PHMB: polyhexamethylene biguanide hydrochloride.

*Specificity not included as these methods are considered the gold standard for the diagnosis of infectious keratitis and theoretically should be 100% in the absence of contamination.

**Table 2:** Advantages and Disadvantages of Culturing Infectious Keratitis

Advantages of Culturing Infectious Keratitis	Disadvantages of Culturing Infectious Keratitis
**Relatively more sensitive in bacterial infections.** **High specificity.** **Guides treatment regimen in refractory cases.** **Prevents treatment failure and/or complications due to wrong initial therapy.** **Shortens exposure to and spending on unnecessary antibiotics.** **Allows for antibiotic sensitivity testing.** **Gives epidemiological data.** **Currently widely available.**	Overall low yield of organisms. Less specific in polymicrobial infection. Less sensitive in fungal, Acanthamoebic, and viral infections. Majority of infections respond to empiric therapy. Takes days-weeks for results, depending on the organism. Rarely changes clinical judgment. Antibiotic sensitivity testing is based on serum concentrations, not corneal. Supply costs, laboratory fees.

## DISCUSSION

**Biological Staining**

Staining corneal samples with Gram and Giemsa stains for immediate diagnostic information shows variable clinical usefulness. Several studies consistently show a low yield of bacteria in bacterial keratitis. Sensitivity of Gram staining was reported for bacterial keratitis to be 36% to 40%, although samples were obtained after antibiotic use which may lower bacterial yield [29]. Other studies have shown the sensitivity of gram staining to be 57% to 67% in similar cases [13, 17, 18]. Staining with potassium hydroxide (KOH) and calcofluor white are only done when there is clinical suspicion of fungi and Acanthamoeba, respectively [30], which would be missed with common staining methods [23].

One study of 3,298 eyes found the sensitivity of KOH wet mount to be 99% for the detection of fungi, 100% for Nocardia, and 91% for Acanthamoeba, suggesting that KOH wet mounts be available and used in all clinics treating infective keratitis [23]. A review reported that staining methods, while showing high specificity, are only able to provide diagnostic value in only 27% to 62% of cases of infectious keratitis and are thus restricted in utility by insufficient sensitivity [30]. Given their variability in sensitivity, staining methods alone do not warrant taking corneal samples in cases of infectious keratitis or pausing antibiotic therapy to increase diagnostic yield. 

**Culturing**

Some published guidelines recommend culturing corneal ulcers in all cases of suspected infectious keratitis due to their potential guidance in cases refractory to empiric therapy [3, 12, 31]. Culturing ideally enables clinicians to identify pathogens that would not respond to empiric therapy (i.e. highly resistant species of bacteria) and switch antimicrobial therapy before ulcers become refractory and progress in severity (Table 2). However, refractivity to medical management is defined as a lack of clinical response in 48 hours after starting empiric therapy [11], and this may be shorter than the time required for culture results to return. For example, anaerobic bacteria need to be incubated up to 10 days for diagnostic reliability [5], and 25% of fungal cultures do not become positive until 2-10 days after inoculation [19]. Furthermore, culturing of the eyelid and conjunctiva has not been particularly helpful in identifying infectious pathogens due to low specificity [32].

Other potential benefits of cultures and sensitivity studies include potentially minimizing patient exposure to antibiotic toxicity, which possibly delays healing of an ulcer [33, 34]. However, culture results were used to guide therapy in only 4% of nonfungal infectious keratitis cases [12]. Moreover, 10% of 82 patients seen by corneal specialists and 0% of 75 patients with corneal ulcers seen at a general ophthalmology practice needed changes in antibiotic regimen due to culture and antibiotic sensitivity results [35]. Since culture results do not often change clinical decision making, their usefulness for preventing medication toxicity remains unclear. 

Cornea specialists have been found to successfully identify between bacterial, fungal, amoebic keratitis for 73% of cases [36] and could distinguish bacterial from fungal origin 66% of the time [37]. This leaves approximately one-third to one-fourth of cases in which culturing may be necessary to guide treatment. Moreover, clinicians could predict Gram stain, genus, and species in only 46%, 25%, and 10% of cases, respectively [37]. Clinicians have shown to more accurately predict causal species in P. aeruginosa and Acanthamoeba infection due to distinguishing characteristics on clinical appearance [36, 38]. These results suggest a need for cultures for microbial identification; however, in a study of 114 mild to severe ulcers, having culture results did not significantly affect outcome [39]. The vast majority (up to 94%) of ulcers resolve with empiric antibiotic therapy [12]. This supports the idea of a selective approach to culturing patients, as the majority of ulcers are successfully treated without knowledge of the causative organism; however, future research is needed to determine which clinical characteristics of ulcers indicate the need for culturing. 

Another argument in the importance of culturing organisms is one of population health, as gathering this information allows us to track which organisms are causing infectious keratitis, their antibiotic resistance profiles, and important risk factors that may emerge to guide future developments in anti-microbial therapy [40]. Along these lines, tailoring antibiotics to avoid the long term use of broad-spectrum antibiotics can help reduce resistance rates [41]. Previous overuse of systemic broad-spectrum antibiotics has resulted in Gram-positive (i.e. methicillin-resistant Staphylococcus aureus [MRSA]) and Gram-negative bacteria resistant to older ophthalmic antibiotics and newer fluoroquinolones used to treat infectious keratitis [41, 42]. However, appointed academic institutions could gather this epidemiological data without the need for general ophthalmologists and non-academic cornea specialists to culture each case of infectious keratitis.

Sensitivity and specificity estimates of cultures in the diagnosis of infectious keratitis are limited by the fact that culturing is currently considered the gold diagnostic standard. Many studies in the literature report percentages of culture-positive cases of suspected infectious keratitis, but the exact proportion of true cases of infectious keratitis picked up on cultures is largely unknown. For example, in a review of four trials, an average of 52% of 735 eyes with ulcerative keratitis were positive for bacteria [40]. However, since other microbial domains cause ulcerative keratitis, as well as noninfectious causes, the sensitivity of culturing bacterial isolates can only be speculated without comparing culturing to other means of detecting bacteria, such as polymerase chain reaction (PCR). 

In a literature review of 20 studies, a median of 50% of clinically diagnosed cases of infectious keratitis were culture-positive [30]. In cases of infectious keratitis due to Acanthamoeba, cultures were positive in only 33%. This is contrasted with 100% and 71% sensitivity of in vivo confocal microscopy (IVCM) and PCR, respectively [28]. In suspected cases of fungal keratitis, culture samples have been reported positive 25% to 59% of the time [24-27]. Viral cultures for HSV have notoriously poor sensitivity. In a study of 48 patients with clinically diagnosed HSV keratitis, only 21% showed positive cultures [20]. Slit lamp examination with fluorescein is often more useful in the diagnosis of viral keratitis. However, the diagnosis may not be made until weeks to months after initial presentation, when bacterial infection can be excluded due to prolonged clinical course, final stromal appearance, and anterior chamber reaction. It is possible that bacterial superinfection can occur on top of viral keratitis. Culturing would be more likely to show bacterial growth in this case, and the treatment of the bacterial infection would take precedence over the viral infection. 

Clearly, the clinical usefulness of microbial cultures in the diagnosis of infectious keratitis is limited by its poor sensitivity in bacterial cases. Culturing becomes even less sensitive in empiric therapy-resistant causes of infectious keratitis (i.e. fungus, Acanthamoeba), which are thought to warrant culturing in the first place (Table 2).

**Financial Limitations**

Staining and culturing corneal samples could abate expensive management of refractory ulcers by preventing complications and unnecessary spending on medications. On the other hand, the cost of supplies and laboratory fees and the lack of technicians trained to expertly interpret smear and stain results on-site may limit their routine use [15]. Infectious keratitis has been reported to have a significant financial burden on patients due to costs associated with clinic visits and medications and indirectly due to lost wages, glasses purchases, and caregiver payments [43]. Patients of lower socioeconomic status (SES) are disproportionately burdened with a higher incidence of infectious keratitis [44, 45], and unnecessary corneal scrapings with subsequent laboratory costs compound this financial burden. However, low SES and uninsured status are independent risk factors for hospitalization from corneal ulceration [46], and therefore may actually warrant the extra expense of cultures. A cost-benefit analysis has not been done to determine if routine sampling would decrease healthcare expenditures by preventing the progression of infectious keratitis.

**Antibiotic Sensitivity Testing**

Besides smears and cultures, corneal specimens can be sent for antibiotic sensitivity testing, which theoretically could be used to guide antibiotic therapy. However, antibiotic sensitivity testing determines susceptibility of bacteria to antibiotics at achievable serum concentrations. Thus, because topical ophthalmic antibiotics can reach much higher concentrations on the cornea, bacteria may respond to treatment for which they were designated resistant [12, 39, 47]. Nine out of 11 patients refractory to empiric therapy should have responded to given antibiotics based on their reported antibiotic sensitivities. Antibiotic sensitivity testing has not been shown to be clinically useful for guiding treatment of infectious keratitis [47].

**Future Alternatives**

Newer technologies are available that can improve our diagnostic ability. Ocular samples can also be sent for PCR, but this technology is not widely available yet in the office setting. PCR has currently been limited to viral keratitis, such as herpetic keratitis [30]. PCR assay can take 4-8 hours to come to a diagnosis compared to 2-10 days for positive bacterial and fungal cultures [19]. Reports comparing PCR to culturing show improved sensitivity and comparable specificities for many different infectious agents, suggesting PCR as another alternative to cultures for the diagnosis of keratitis [28, 30]. In vivo confocal microscopy has been reported with high sensitivity in the diagnosis of larger organisms causing infectious keratitis, specifically Acanthamoeba and filamentous fungi [31, 48]. 

## CONCLUSION

When faced with a case of infectious keratitis, ophthalmologists must rely on accrued experience and clinical acumen to determine whether cultures will help guide treatment and improve prognosis. Stains and cultures have the potential to guide future therapy for a patient should the ulcer resist empiric therapy. If conducted promptly, smears and cultures may even prevent progression of infectious keratitis and save the patient from vision loss and expensive medical bills. Furthermore, gathering epidemiological data on infectious keratitis could help develop future antimicrobial therapy. Despite these, staining methods and cultures are especially limited by poor sensitivity in microbes that cause treatment failure, often take too long to yield diagnostic results, and have not been shown to frequently change clinical decision making in the community-based setting. Newer technologies, such as PCR, may increase diagnostic yield from corneal scrapings but are not yet widely available. Currently, there is a shortage of well-designed studies evaluating the clinical utility and cost-effectiveness of accepted guidelines for the diagnosis of infectious keratitis. 

## DISCLOSURE

Ethical issues have been completely observed by the authors. All named authors meet the International Committee of Medical Journal Editors (ICMJE) criteria for authorship of this manuscript, take responsibility for the integrity of the work as a whole, and have given final approval for the version to be published. No conflict of interest has been presented.

## Funding/Support:

Research to Prevent Blindness, NY, USA
